# From Molecules to Imaging: Assessment of Placental Hypoxia Biomarkers in Placental Insufficiency Syndromes

**DOI:** 10.3390/cells12162080

**Published:** 2023-08-17

**Authors:** Fatimah M. Al Darwish, Lotte Meijerink, Bram F. Coolen, Gustav J. Strijkers, Mireille Bekker, Titia Lely, Fieke Terstappen

**Affiliations:** 1 Department of Biomedical Engineering and Physics, Amsterdam Cardiovascular Sciences, Amsterdam UMC, University of Amsterdam, 1105 AZ Amsterdam, The Netherlands; b.f.coolen@amsterdamumc.nl (B.F.C.); g.j.strijkers@amsterdamumc.nl (G.J.S.); 2 Department of Obstetrics, Wilhelmina Children’s Hospital, University Medical Center Utrecht, Utrecht University, 3584 EA Utrecht, The Netherlands; l.meijerink-10@umcutrecht.nl (L.M.); m.n.bekker-3@umcutrecht.nl (M.B.); t.lely@umcutrecht.nl (T.L.); f.terstappen@umcutrecht.nl (F.T.)

**Keywords:** placental hypoxia, molecular biomarkers, non-invasive imaging

## Abstract

Placental hypoxia poses significant risks to both the developing fetus and the mother during pregnancy, underscoring the importance of early detection and monitoring. Effectively identifying placental hypoxia and evaluating the deterioration in placental function requires reliable biomarkers. Molecular biomarkers in placental tissue can only be determined post-delivery and while maternal blood biomarkers can be measured over time, they can merely serve as proxies for placental function. Therefore, there is an increasing demand for non-invasive imaging techniques capable of directly assessing the placental condition over time. Recent advancements in imaging technologies, including photoacoustic and magnetic resonance imaging, offer promising tools for detecting and monitoring placental hypoxia. Integrating molecular and imaging biomarkers may revolutionize the detection and monitoring of placental hypoxia, improving pregnancy outcomes and reducing long-term health complications. This review describes current research on molecular and imaging biomarkers of placental hypoxia both in human and animal studies and aims to explore the benefits of an integrated approach throughout gestation.

## 1. Introduction

**Figure 1:**
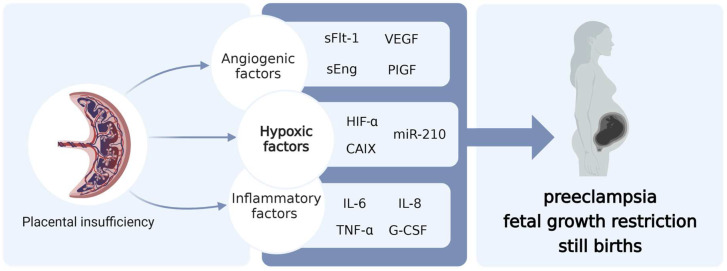
Interplay of factors contributing to placental insufficiency: Examples of potential biomarkers related to each factor presented in the blue box. sFlt-1: Soluble fms-like tyrosine kinase-1, VEGF: Vascular endothelial growth factor, sEng: Soluble endoglin, PIGF: Placental growth factor, HIF-α: Hypoxia-inducible factor–alpha, CAIX: Carbonic Anhydrase IX, miR-210: microRNA-210, IL6: Interleukin 6, IL8: Interleukin 8, TNF-α: Tumor necrosis factor-alpha, G-CSF: Granulocyte colony-stimulating factor.

Placental insufficiency is associated with pregnancy complications, including pre-eclampsia (PE), fetal growth restriction (FGR), and stillbirth [1]. The pathophysiology of placental insufficiency is not entirely clear. It involves many factors, including anti-angiogenic [2,3], pro-inflammatory [4,5], and hypoxic factors [6], representing a complex web of interactions that profoundly affects the delicate balance required for optimal fetal development (Figure 1).

Placental hypoxia is a central factor in the development of placental insufficiency [7]. During placentation, the trophoblast cells differentiate and invade the maternal tissues, establishing the vital placental vascular network that facilitates nutrient and gas exchange between the mother and fetus. Any disruption in this process can lead to inadequate oxygen supply to the placenta and subsequently contribute to placental insufficiency and related pregnancy complications [1,7]. Therefore, assessing placental oxygenation or hypoxia is essential for understanding part of the pathophysiology of placental insufficiency.

The current approach to monitoring placental function involves ultrasound biometry, Doppler measurements, and cardiotocography to assess fetal wellbeing [8]. Despite their utility, the current biomarkers fall short in detecting early signs of placental insufficiency and precisely predicting adverse pregnancy outcomes. Approximately half of FGR cases remain undetected during pregnancy, leading to missed opportunities for timely interventions. Furthermore, once FGR is detected, existing monitoring strategies may inadequately identify fetal compromise in a timely and reliable manner [9]. Therefore, the incorporation of reliable biomarkers becomes imperative, especially in determining the optimal timing for intervention and delivery, a crucial consideration given the delicate balance between the risks of stillbirth and those associated with preterm birth [10].

One way to identify placental hypoxia is the use of molecular biomarkers, which can be detected in maternal blood during pregnancy or in placental tissue after delivery. Circulating biomarkers in maternal blood can provide valuable information about the overall health status. On the other hand, ex vivo investigations of placental tissue can be more specific, as it allows the direct examination of the placenta; however, it provides only a static representation of placental function at a specific time, disregarding the dynamic changes in placental development throughout gestation. Nevertheless, the assessment of the placenta after delivery becomes apparent too late to intervene, underscoring the importance of alternative methods to monitor placental health in real-time.

The assessment of dynamic changes of the in utero condition can be facilitated by non-invasive imaging techniques that can safely and effectively assess the placental and fetal condition over time. Non-invasive imaging offers a more comprehensive understanding of the underlying biological processes that drive placental disease development and progression during multiple gestational phases. Fortunately, recent advances in imaging technologies such as photoacoustic imaging (PAI) and magnetic resonance imaging (MRI) have shown great promise in detecting and monitoring placental hypoxia [11,12]. These imaging techniques have the potential to complement molecular biomarkers, offering a more thorough understanding of placental function throughout gestation.

While several studies have individually investigated the utility of molecular and imaging biomarkers, there is an emerging imperative to establish a connection between these two approaches. In this narrative review, we therefore aimed to examine the current research on molecular and imaging biomarkers in human and animal studies and identify studies that have adopted an integrated approach of incorporating both biomarker types to assess placental hypoxia.

## 2. Molecular Biomarkers of Hypoxia

Molecular biomarkers of hypoxia are proteins and genes that are activated in response to reduced oxygen levels. Several molecular biomarkers, including HIF-1α, CAIX, and miR-210, are involved in the cellular response to hypoxia [13,14,15]. These biomarkers can be measured in placental tissues or maternal blood samples using various techniques, such as immunohistochemistry, ELISA, and qPCR. Elevated levels of these biomarkers have been associated with placental hypoxia and adverse pregnancy outcomes. It is important to note that there are additional downstream biomarkers, particularly angiogenic factors, that provide further insight into vascular and endothelial dysfunction [2]. However, discussing these factors falls beyond the scope of this narrative review, which focuses specifically on hypoxia.

### 2.1. Hypoxia Inducible Factor (HIF)-α

Hypoxia Inducible Factor (HIF)-α is a transcription factor that plays a critical role in the cellular response to hypoxia or low oxygen levels. It regulates the expression of genes involved in various physiological processes, including angiogenesis, metabolism, and cell survival [16]. HIF is composed of two subunits: HIF-α and HIF-β. The HIF-α subunit is highly regulated by oxygen availability and it degrades rapidly under normoxia, while it stabilizes under hypoxia. Under hypoxic conditions, the stabilization of HIF-α results in its accumulation. Subsequently, HIF-α binds with HIF-β to form an active HIF complex, which triggers the transcription of target genes, including VEGF. This process aims to enhance the delivery of oxygen to the hypoxic region of tissues [1]. To gain insights into the significance of HIF-α in placental insufficiency, several studies have explored its expression and activity in both human and animal models.

#### 2.1.1. Human Studies

During the first trimester of pregnancy, the levels of HIF-1α protein in the maternal blood serum, determined through ELISA testing, have demonstrated effectiveness in predicting preeclampsia (PE). Without the use of uterine artery Doppler, the sensitivity and specificity of HIF-1α levels were found to be 66.7% and 71.5%, respectively. When combined with Doppler, the sensitivity increased slightly to 74.2%. This study concludes that the measurement of HIF-1α protein levels using ELISA, either alone or in combination with uterine artery Doppler, is an effective approach for predicting PE during the first trimester of pregnancy [17]. Tianthong et al. showed that serum HIF-1α levels can distinguish between early-onset and late-onset PE, with higher expression observed in early-onset cases compared to late-onset and normal control samples [17]. In another prospective longitudinal study in high cardiovascular risk pregnancies, HIF-1α mRNA levels in plasma were able to predict later-stage development of PE, with elevated levels observed until 23 weeks of gestation [18]. Others also showed that HIF-1α expression is elevated in prenatal blood samples taken at the diagnosis of PE, as well as in postnatal blood samples from pregnancies affected by PE when compared to control samples [19]. After delivery, studies conducted on ex vivo placental tissue have consistently demonstrated elevated levels of HIF-1α and HIF-2α in pregnancies affected by PE and FGR [20,21,22,23,24,25]. Nevertheless, the number of studies simultaneously examining both HIF-1α and HIF-2α is limited, but the available research suggests that HIF-2α, rather than HIF-1α, is prominently induced in placentas from patients with FGR and PE [26,27]. Comparing to blood samples, ex vivo analysis of HIF-1α has not been successful in differentiating between early-onset and late-onset PE [25]. This suggests that although HIF-1α levels are altered in ex vivo placenta samples of PE, they may not be a suitable standalone marker for discriminating between early and late-onset PE cases.

Furthermore, elevated HIF-1α levels were recorded in pregnant women living in high altitudes, which is associated with an increased risk of PE and FGR [28,29]. Analysis of placental tissue from women residing at high altitudes (>2700 m) revealed increased HIF-1α mRNA and protein levels, suggesting the activation of the hypoxia-inducible factor pathway in response to chronic hypoxia. This upregulation of HIF-1α is likely to facilitate placental oxygen transport by promoting erythropoiesis and placental angiogenesis, which are critical for supporting fetal growth and development under low oxygen conditions [28]. These findings provide further evidence of the impact of placental hypoxia on pregnancy outcomes and highlight the relevance of the high-altitude model in understanding the molecular mechanisms underlying FGR and PE.

#### 2.1.2. Animal Models

In animal models of placental insufficiency, HIF-α expression has been primarily assessed ex vivo in placental tissue. These studies have consistently reported increased levels of HIF-1α mRNA in various models of placental insufficiency, including modified Reduced uterine perfusion pressure (RUPP) (created by ligation of the ovarian arteries only) in mice [30], L-NAME rats (treated with Nω-nitro-L-arginine methyl ester) [31], and rats on a low-sodium diet [32]. Additionally, elevated HIF-1α protein levels have been observed in different models such as RUPP rats and mice [16,33,34,35,36], L-NAME rats and mice [31,37], LPS-treated rats [38], and hypoxia-induced models in rats and mice [16]. In the case of both modified RUPP and selective RUPP (which involves the ligation of both ovarian and uterine arteries), elevated HIF-2α mRNA levels have been found compared to sham. However, HIF-1α was found to be reduced specifically in the case of selective RUPP [39].

These findings suggest that measurements of HIF-1α and HIF-2α hold promise as potential biomarkers for the prediction and identification of placental insufficiency. However, further research is needed to fully understand their clinical significance and effectiveness in predicting conditions such as PE, FGR and stillbirth. Additionally, exploring the combination of these biomarkers with other relevant markers to develop a robust prediction model is essential.

### 2.2. Carbonic Anhydrase IX (CAIX)

Carbonic Anhydrase IX (CAIX) is a transmembrane protein that belongs to the carbonic anhydrase family and plays a crucial role in the regulation of acid-base balance in cells and highly expressed in hypoxic conditions [40]. In normal condition, CAIX is expressed in various tissues, including the gastrointestinal tract, kidney, and reproductive organs. In the placenta, CIAX expression can be observed in the villous cytotrophoblast during early gestation, indicating its involvement in the placental structure and function at this stage. Additionally, CIAX is consistently present in the chorionic plate mesenchymal cells throughout the entirety of a healthy gestation [41]. In placental insufficiency, CAIX have primarily been investigated in clinical research studies.

#### Human Studies

CAIX serum levels have been investigated as a potential biomarker for predicting the onset of pregnancy complications. A recent longitudinal study has examined the possibility of using CAIX serum level as a biomarker to predict the onset of pregnancy complications in women with a higher risk of developing PE [13]. The study found that CAIX levels were significantly increased in women who later developed PE, starting from the 28th week of gestation, with a high level of sensitivity and specificity (100% and 81.82%, respectively) [13]. This suggests that CAIX could be a reliable predictive biomarker for the onset of PE. Also, CIAX serum level was elevated in PE patients during the third trimester compared to normal pregnancies [42]. In addition, in a study comparing oxidative stress and hypoxia markers in HELLP syndrome, a subtype of severe PE, CAIX levels were significantly higher in HELLP syndrome patients [43]. These findings suggest that hypoxia induces CAIX and plays a significant role in the pathophysiology of these pregnancy complications.

### 2.3. miR-210

MicroRNAs (miRNAs) are a type of small non-coding RNA that play a critical role in regulating gene expression at a posttranscriptional level and they function as a regulator of cell activities including growth, differentiation and apoptosis. Thus, they can serve as molecular biomarkers for various pathological conditions. Among the many miRNAs identified, miR-210 has emerged as a promising hypoxia biomarker in many diseases including cardiovascular diseases, cancer and PE hypoxia [44,45]. Overexpression of miR-210 appears to negatively impact cell migration and trophoblast invasion, which are crucial for normal placental development [45].

#### 2.3.1. Human Studies

Overexpression of miR-210 has been detected in the plasma of PE patients [46,47,48]. In the second trimester (12–14 weeks of gestation), miR-210 was upregulated in the plasma of pregnant women who later developed PE [14]. Later in pregnancy, both severe (sPE) and mild PE (mPE) patients exhibited significantly higher plasma miR-210 expression levels compared to controls [46,47]. Notably, a study investigating maternal exosomal miR-210 levels demonstrated that the highest expression was observed in sPE, followed by mPE, while there were no significant differences between gestational and chronic hypertension compared to normal pregnancy. This finding emphasizes the role of a hypoxic placenta in PE development [47]. Furthermore, a PCR array of miR-210 target genes revealed that downregulated genes in both severe and mild PE cases were associated with immunosuppression, apoptosis, cell growth, signaling, angiogenesis, and DNA repair which can be associated with PE risk and severity [46]. In addition, studies have shown that miR-210 is upregulated in ex vivo placental tissue of patients with PE, both with and without small-for-gestational age neonates [49]. A recent review reported upregulation in the placental tissue of PE patients in the majority of studies but downregulation in one study, suggesting inconsistency of the results [48].

#### 2.3.2. Animal Studies

In a mouse model of PE induced by toll-like receptor 3 agonist, miR-210 was upregulated in the placenta, which is partly driven by HIF-1α and nuclear factor κB (NF-κB) [50]. The expression level of miR-210 was upregulated in the uterine arteries of sheep exposed to high altitude hypoxia [51]. These results imply that miR-210 is a promising molecular biomarker for placental hypoxia in PE. It has the potential of early prediction of the disease. Its expression in the placenta and plasma of PE patients, as well as its impact on downstream gene expression, suggest its potential utility in clinical practice.

## 3. Imaging Biomarkers of Hypoxia

Imaging biomarkers are quantitative or qualitative measurements or features derived from images that provide information about biological processes or functions of a tissue or organ. These biomarkers can be used to assess disease progression, monitor treatment response, and aid in diagnosis. In pregnancy, direct and dynamic measures of placental function are possible through non-invasive imaging techniques such as MRI and photoacoustic imaging (PAI) [11,12].

### 3.1. T2* MRI

MRI has emerged as a non-invasive tool to assess placental function in vivo. Among various MRI sequences, T2* weighted MRI has shown promising results as an imaging biomarker of placental hypoxia [52]. T2* is a magnetic resonance imaging parameter that reflects the decay of the MRI signal intensity over time due to factors such as magnetic field inhomogeneities caused by the presence of substances like deoxygenated hemoglobin in the tissue [53]. This sensitivity to changes in the local magnetic field makes T2* MRI useful as an indicator of tissue hypoxia. Changes in T2* values can provide valuable insights into alterations in oxygenation levels within the tissue, making it a critical imaging biomarker for studying conditions like placental hypoxia.

Baseline quantitative T2* values can be directly related to the oxygenation status of the tissue. Based on T2* MRI, two functional parameters have been used to assess the change in signal intensity in response to a gas challenge, such as hyperoxia or hypercapnia. One is delta T2*, which is based on quantitative T2* values, and this parameter estimates the change in placenta T2* value from two T2* scans obtained at different conditions. The other parameter is Blood Oxygen Level Dependent (BOLD) MRI, which is a relative measure of T2* signal change over time in response to a challenge. Delta T2* and delta BOLD under gas challenge reflects the hemodynamic status of the tissue.

#### 3.1.1. Human Studies

In a prospective observational study of 100 singleton pregnancies, placental T2* was found to predict low birth weight better than uterine artery Doppler [54]. Placental T2* was also able to distinguish between FGR and small-for-gestational-age (SGA) fetuses, both of which were lower than the control group [55]. Additionally, placental T2* values were significantly lower in FGR cases validated with ex vivo placental pathology showing necrosis and fibrosis [56]. Moreover, in a cohort of SGA fetuses with normal fetal Doppler flows, T2*-weighted placental MRI was found to be a significant predictor of placenta-related outcomes such as placental vascular malperfusion, SGA at birth, PE, and preterm delivery [57]. A recent large prospective longitudinal study of 316 pregnancies shows the potential of T2* to identify pregnancies at risk starting from early to mid-gestation (10–20 weeks) [58].

Limited studies used oxygenation challenge with MRI to assess the placental condition in abnormal pregnancies. In cases diagnosed with FGR, the peak delta R2* (1/T2*) value, representing a change in oxygen saturation levels, during hyperoxia was lower in FGR cases compared to healthy controls [59]. However, another study found no difference in delta T2* values in the placenta [60]. These findings indicate that further research is needed to better understand the relationship between placental oxygenation and abnormal pregnancies, as well as to determine the reliability and consistency of oxygenation challenge with MRI as a diagnostic tool.

#### 3.1.2. Animal Studies

In a mouse model of PE induced by fetoplacental overexpression of STOX1A, a transcription factor, MRI revealed a reduction in T2* of the PE placentas [61]. Similarly, in a cohort of pregnant Rhesus macaques, placental oxygenation assessed by T2* was shown to be reduced [62]. While in the model of FGR induced by ligation of left uterine vascular pedicle, baseline T2* was not different between FGR placentas and control, however, employing BOLD MRI and evaluating delta T2*, a significant decrease in delta T2* in response to hyperoxygenation was observed in FGR placentas compared to controls [63,64]. Similarly, BOLD-MRI showed impaired placental responses to hypercapnia in a hypoxic mouse model of placental insufficiency [65].

### 3.2. Photoacoustic Imaging (PAI)

PAI is a novel imaging technique that combines optical and ultrasound imaging using non-ionizing nanosecond pulses [66]. This technique can measure placental oxygenation directly as oxyhemoglobin and deoxyhemoglobin absorb light at different wavelengths. With this information, it becomes possible to calculate the oxygen saturation levels and makes. PAI is a promising technique for placental insufficiency particularly due to its ability to provide absolute measurements of placental oxygenation, which allows for objective tracking of trends in oxygenation throughout gestation. However, its use in humans is hampered by the imaging depth limit, which is, however, an area of ongoing development [66]. Therefore, the application of PAI in pregnancy research is currently limited to animals.

#### Animal Studies

During the transitional phase towards clinical utility, PAI already has been proven a valuable tool for evaluating placental and fetal oxygenation in animal models. Several studies reported a reduction in placental oxygen saturation (sO2) measured by PAI in response to placental hypoxia induced by L-NAME treatment or ACE2 knockout followed over time [35,37]. Additionally, longitudinal assessment of placental oxygenation revealed a reduction followed by a slight recovery over time in placental sO2 in the RUPP rat model [35]. Using the RUPP model of placental insufficiency, PAI has demonstrated promising potential in testing the therapeutic response on placental oxygenation [67]. In addition, the reduced placental oxygenation detected by PAI was validated with tissue hypoxia biomarkers, HIF-1α, confirming placental hypoxia [35,67].

## 4. Discussion: The Integration of Molecular and Imaging Biomarkers and Its Challenges

**Figure 2:**
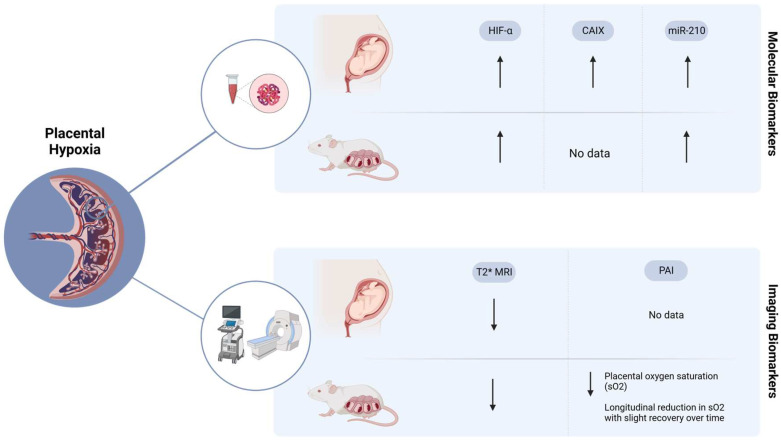
Overview of the molecular and imaging biomarkers of placental hypoxia. HIF-α [16,17,18,19,20,21,22,23,24,25,28,30,38], CAIX [13,42,43], miR-210 [14,46,47,48,49,50], T2* MRI [16,55,56,59,62,63,64], PAI [35,37,67].

Placental insufficiency in pregnancy complications such PE and FGR can be better understood through the examination of biomarkers. These biomarkers are associated with key factors involved in the pathophysiology of placental insufficiency, with a particular focus on hypoxia in this review. The studies reviewed consistently demonstrate elevated levels of HIF-α in cases of PE and FGR, both in human blood samples and placental tissue, suggesting their potential as biomarkers for predicting and identifying placental insufficiency. Alongside HIF-α, CAIX and miR-210 have also emerged as promising biomarkers for placental hypoxia. Clinical studies have shown significant increases in CAIX serum levels in women who later developed PE, while miR-210 expression has been detected in plasma and tissue samples from patients. These findings underscore the potential value of these biomarkers in improving our understanding and detection of placental hypoxia in pregnancy complications (Figure 2).

Imaging biomarkers, including T2* MRI and PAI, provide valuable non-invasive approaches to evaluate placental function. T2* MRI shows promise in identifying pregnancies affected by placental insufficiency, enabling the prediction of low birth weight and differentiation between FGR and small-for-gestational-age fetuses. Although PAI is currently limited to animal studies, it has the potential in assessing placental and fetal oxygenation (Figure 2). To maximize the potential of non-invasive imaging biomarkers, it is crucial to utilize them longitudinally over time, offering more detailed insights into the condition of the feto-placental unit. While Doppler ultrasound has been widely used, there is a need to introduce new diagnostic tools that harness the potential of more sensitive and direct imaging techniques. Despite the current challenges associated with PAI in human applications, such as limited depth penetration and safety concerns [66], ongoing technological advancements and research efforts aim to overcome these limitations and enhance its clinical utility.

Studying molecular or imaging biomarkers solely has established consistent findings in associating placental hypoxia with an increase in molecular biomarkers such as HIFa, CAIX, and miR-210 levels or a decrease in imaging biomarker values obtained by T2* MRI and PAI. On the other hand, few preclinical studies using PAI have explored the integration of molecular and imaging biomarkers to validate their findings. For example, oxygen saturation values were compared to ex vivo HIF-α levels in placental tissue, revealing a negative correlation between the two [35,37,67]. This suggests that the levels of oxygen saturation and HIF-α expression may provide complementary information about placental hypoxia. Clinical studies have demonstrated that ex vivo pathological examinations of affected placentas reveal abnormalities associated with vascular malperfusion, which are consistent with the outcomes obtained from T2* MRI within the same study [54,56,68]. However, it is important to note that, to our knowledge, no clinical studies have integrated hypoxia molecular biomarkers with imaging biomarkers in the assessment of placental insufficiency. The integration of these biomarkers in future clinical studies holds great potential for a more comprehensive and accurate evaluation of placental hypoxia.

However, the integration of molecular and imaging biomarkers in detecting placental hypoxia presents certain challenges that need to be addressed. One major challenge is the standardization of biomarker assessment across different imaging facilities and laboratories to ensure consistent and reliable results. Additionally, the assessment of both molecular and imaging biomarkers within the same study can be time-consuming. However, this approach is crucial to avoid potential false assumptions that may arise when connecting the results of molecular biomarkers from one study with imaging biomarkers from another study conducted under different conditions. By integrating both types of biomarkers in a single study, we can enhance the accuracy and reliability of placental hypoxia detection.

## 5. Conclusions

Based on this review, HIF-α as the molecular biomarker and T2* MRI as the imaging technique hold promise for predicting and identifying placental-insufficiency-related conditions. Together, they may provide a comprehensive approach to assess placental function and guide interventions. Nonetheless, further research and validation studies are warranted to fully elucidate the clinical significance and effectiveness of this molecular biomarker and imaging technique pair in improving maternal and fetal outcomes in cases of placental insufficiency.

The integration of molecular and imaging biomarkers has the potential to revolutionize the detection and monitoring of placental hypoxia. However, the current utilization of this integrated approach remains limited to a few studies. To fully understand the clinical utility of this approach, future investigations should consider several key factors. These include evaluating biomarkers at early gestational ages, conducting assessments at multiple time points, examining well-defined patient populations, and utilizing preclinical animal models. By addressing these factors, researchers can thoroughly investigate the potential benefits of this approach and validate their clinical utility in the management of placental insufficiency by finding the optimal indication for timing of pre-term deliveries.
